# Upregulation of LINC01426 promotes the progression and stemness in lung adenocarcinoma by enhancing the level of SHH protein to activate the hedgehog pathway

**DOI:** 10.1038/s41419-021-03435-y

**Published:** 2021-02-10

**Authors:** Xiaoli Liu, Zuwei Yin, Linping Xu, Huaimin Liu, Lifeng Jiang, Shuochuan Liu, Xu Sun

**Affiliations:** 1 Affiliated Cancer Hospital of Zhengzhou University & Henan Cancer Hospital, Zhengzhou, China; 2 Tsinghua University, Beijing, China; 3 Queen Mary College of Medicine, Nanchang University, Nanchang, China

**Keywords:** Lung cancer, Cell biology

## Abstract

Long noncoding RNAs (lncRNAs) play crucial roles in regulating a variety of biological processes in lung adenocarcinoma (LUAD). In our study, we mainly explored the functional roles of a novel lncRNA long intergenic non-protein coding RNA 1426 (LINC01426) in LUAD. We applied bioinformatics analysis to find the expression of LINC01426 was upregulated in LUAD tissue. Functionally, silencing of LINC01426 obviously suppressed the proliferation, migration, epithelial–mesenchymal transition (EMT), and stemness of LUAD cells. Then, we observed that LINC01426 functioned through the hedgehog pathway in LUAD. The effect of LINC01426 knockdown could be fully reversed by adding hedgehog pathway activator SAG. In addition, we proved that LINC01426 could not affect SHH transcription and its mRNA level. Pull-down sliver staining and RIP assay revealed that LINC01426 could interact with USP22. Ubiquitination assays manifested that LINC01426 and USP22 modulated SHH ubiquitination levels. Rescue assays verified that SHH overexpression rescued the cell growth, migration, and stemness suppressed by LINC01426 silencing. In conclusion, LINC01426 promotes LUAD progression by recruiting USP22 to stabilize SHH protein and thus activate the hedgehog pathway.

## Introduction

Lung adenocarcinoma (LUAD) is a subtype of non-small cell lung cancer (NSCLC), which has become the most common cancer of tumor-related deaths all over the world^1–3^. Metastasis occurs in most of the LUAD patients at advanced stage^4^. Although advances have been made in the diagnosis and therapy, the prognosis and overall survival of LUAD patients remain unoptimistic^5^. Thus it is urgent to explore potential molecular mechanisms underlying LUAD progression to find novel therapeutic targets for LUAD patients.

Long noncoding RNAs (lncRNAs) are characterized as a class of transcripts with lengths of more than 200 nucleotides and without protein-coding ability^6,7^. Accumulating evidence has indicated that lncRNAs are involved in the regulation of various cellular processes. For example, lncRNA NEAT1 acts as a miR-196a-5p sponge to promote colorectal cancer cell proliferation and migration^8^. LncRNA B3GALT5-AS1 inhibits colon cancer metastasis via targeting miR-203^9^. LncRNA EWSAT1 acts as a sponge of miR-326/-330-5p to increase human nasopharyngeal carcinoma cell growth^10^. Recent reports have displayed that numerous lncRNAs, including lncRNA DGCR5^11^, lncRNA HOXA11-AS^12^, and lncRNA NEAT1^13^ are associated with LUAD. Here, we investigated the molecular mechanism of a novel lncRNA long intergenic nonprotein coding RNA 1426 (LINC01426) in LUAD. LINC01426 has been reported to be a tumor promoter in glioma^14^ and differentially expressed in squamous cell lung carcinoma^15^. However, it is unknown whether LINC01426 exerts the same functions in LUAD. Therefore, we investigated its functions in LUAD.

It has been reported that lncRNAs function through multiple pathways, including IGF pathway^16^, hedgehog pathway^17^, ERK pathway^18^, and AKT pathway^19^. In our study, we explored the downstream signaling pathway of LINC01426 in LUAD cells.

SHH is a type of protein in the hedgehog pathway and has been reported to take part in the progression of disease^20^. In our study, we detected the relationship between LINC01426 and SHH in LUAD cells.

As reported previously, USP22 promotes the proliferation of non-small cell lung cancer by regulating the ubiquitination of COX-2^21^. USP22 also regulates EGFR ubiquitination in lung adenocarcinoma^22^. GSK3β promotes glioblastoma tumorigenesis through inducing KDM1A deubiquitination by USP22^23^. In our study, we also investigated whether USP22 was involved in the LINC01426-mediated hedgehog pathway.

Taken together, we aimed at exploring the functions of LINC01426 in LUAD and its regulatory mechanism with the hedgehog pathway.

## Materials and methods

### Cell lines and reagents

Human bronchial epithelial cell line (HBE) and human LUAD cell lines (H1299, A549, PC-9, Calu3) used in this study were procured from ATCC (Manassas, VA). They were grown with 10% FBS and 1% antibiotics in DMEM (Invitrogen, Carlsbad, CA) under 37 °C and 5% CO_2_. To treat PC-9 and Calu3 cells, 5 μg/ml of cycloheximide (CHX), 20 mmol/l of LiCl were available from Sigma-Aldrich (St. Louis, MO). A total of 20 mg/ml of MG132 and 100 μM of NSC 228155, 50 μM of Honokiol were purchased from Selleck Chemicals (Houston, TX). Totally, 10 nM of IGF-1 was bought from PeproTech (Rocky Hill, NJ). Totally, 10 nM of SAG was obtained from Abcam (Cambridge, MA).

### Total RNA isolation and qRT-PCR

Total RNA isolation from cultured cells was complete using Trizol reagent (Invitrogen), then cDNA was synthesized by PrimeScript^™^ RT reagent kit as guided (Takara, Shiga, Japan). Gene expression was evaluated by qRT-PCR with SYBR Premix Ex Taq II (Takara) and calculated based on the 2^−ΔΔCt^ method. GAPDH or U6 served as the reference gene.

### Subcellular fractionation assay

1 × 10^6^ PC-9 and Calu3 cells were collected, then centrifuged and washed in phosphate-buffered saline (PBS). PARIS^™^ was applied to perform subcellular fractionation assay as required (Invitrogen). LINC01426 content was examined in the cell cytoplasm and cell nucleus with qRT-PCR.

### Fluorescence in situ hybridization

Cells fixed by 4% formaldehyde were rinsed in PBS and digested, then dehydrated and hybridized with LINC01426-FISH probe (Ribobio, Guangzhou, China). The cell nucleus was counterstained by DAPI solution and visualized by fluorescence microscope (Olympus Corp., Tokyo, Japan).

### Transfection

The short hairpin RNAs (shRNAs) specifically targeting LINC01426 or USP22 were synthesized by GenePharma (Shanghai, China) and transfected into cancer cells using Lipofectamine 2000 (Invitrogen). The full-length cDNA sequence of SHH was inserted into the pcDNA3.1 vector (Invitrogen) for overexpression. All transfections were conducted for 48 h.

### Colony formation assay

PC-9 and Calu3 cells were cultured for 14 days in 6-well plates at a density of 500/well. After that, cells were fixed in 4% paraformaldehyde, stained with 0.1% crystal violet, and finally counted manually.

### EdU staining

Cells were seeded in 96-well plates at 5 × 10^4^ density for incubation with EdU medium diluent and fixed for 2 h. Cell proliferation was assayed based on the protocol of the EdU staining reagent (Ribobio). After washing in PBS, cells were cultured in DAPI solution, then observed under a fluorescence microscope.

### Flow cytometry analysis

The transfected cells (2 × 10^5^) were harvested and rinsed in precooled PBS, then treated with Annexin V-FITC/PI Apoptosis kit as per user manual (BD Biosciences, San Jose, CA). After double-staining in binding buffer, a flow cytometer was used based on the protocol (BD Biosciences).

### TUNEL staining

Terminal deoxynucleotidyl transferase dUTP nick end labeling (TUNEL) staining reagent was available from Beyotime (Haimen, China) to detect apoptotic cells. After fixing and washing, cells were permeabilized in 0.1% TritonX-100 for staining with TUNEL kit and DAPI solution, then examined using a fluorescence microscope.

### JC-1 staining

After 5 min of centrifugation, the processed cells in 96-well plates were treated with JC-1 staining reagent (Beyotime) for 30 min. Following washing in assay buffer, mitochondrial transmembrane potential (ΔΨm) was monitored by a fluorescent plate reader.

### Transwell migration assay

Migration assay was completed using the Transwell chamber (8-mm pore size; Corning, Corning, NY). The lower chamber was filled with 100% medium, and cells re-suspended in serum-free medium were seeded in the upper chamber for 24 h. Then, cells migrated to the bottom surface were fixed and stained by crystal violet dye. Five fields were randomly selected for counting with a microscope.

### Western blot

Total protein samples were isolated using radioimmunoprecipitation assay lysis buffer and separated on 12% sodium dodecyl sulfate-polyacrylamide gel electrophoresis, then transferred to polyvinylidene fluoride membranes and cultured with 5% nonfat milk. After that, the primary antibodies against N-cadherin, E-cadherin, NANOG, OCT4, SHH, DHH, IHH, Gli1, Gli2, USP22, and GAPDH (internal reference), as well as secondary antibodies labeled by horseradish peroxidase, were all procured from Abcam (Cambridge, MA) and used to incubate membranes. Signals of protein bands were detected using an ECL system (Bio-Rad, Hercules, CA).

### Immunofluorescence staining (IF)

Cells were cultured for 24 h on culture slides and washed in PBS after adhering to slides. Then, the fixed cells were treated with 5% bovine serum albumin and incubated with primary antibodies specific to E-cadherin and N-cadherin. Following washing, secondary antibody was added, the slides were stained in DAPI and examined by Olympus microscope.

### Sphere formation assay

Cell lines were cultured with sphere medium in the 96-well ultralow attachment plates in line with instruction (Corning) for 7 days, with 10 cells in each well. Cell clusters with a diameter >50 mm were seen as sphere cells and counted.

### Co-immunoprecipitation (Co-IP)

Cells were treated with IP lysis buffer, then lysates were probed with SHH or USP22 antibody and magnetic beads at 4 °C all night. After that, samples were washed in PBS and eluted for western blot.

### RNA pull-down assay

Pierce Magnetic RNA-Protein Pull-Down Kit was available from Thermo Fisher Scientific (Waltham, MA) and used as instructed. Cell lysates were mixed with biotinylated RNA probes for LINC01426 and its antisense RNA (LINC01426 AS), then added with magnetic beads for 1 h. After elution, a western blot assay was performed to detect the recovered proteins.

### RNA immunoprecipitation (RIP) assay

After culturing in RIP lysis buffer, lysates from processed cells were conjugated with USP22 antibody or control IgG antibody in magnetic beads. After RNA enrichment, qRT-PCR was implemented to analyze RNA enrichment.

### Tumor xenograft

The Animal Research Committee of Affiliated Cancer Hospital of Zhengzhou University approved the protocols for animal experiments. The mice (4-week-old BALB/c nude female mice, 5 per group) were subcutaneously injected with PC-9 cells stably transfected with sh/Ctrl, sh/LINC01426#1, and sh/LINC01426#1+SHH, and every four days the tumor volume was recorded. The mice were all sacrificed on the 28th day and tumor tissues were collected to measure tumor weight.

### Statistical analysis

The experiments in our study were all conducted in triplicate, results were given as the means ± standard deviation (S.D.) and analyzed using PRISM 6 as guided (GraphPad, San Diego, CA). The statistical analysis was performed using Student’s *t* test or one‐way (two-way) ANOVA. The significant level was set as p-values below 0.05.

## Results

### LINC01426 is overexpressed in LUAD

**Fig. 1:**
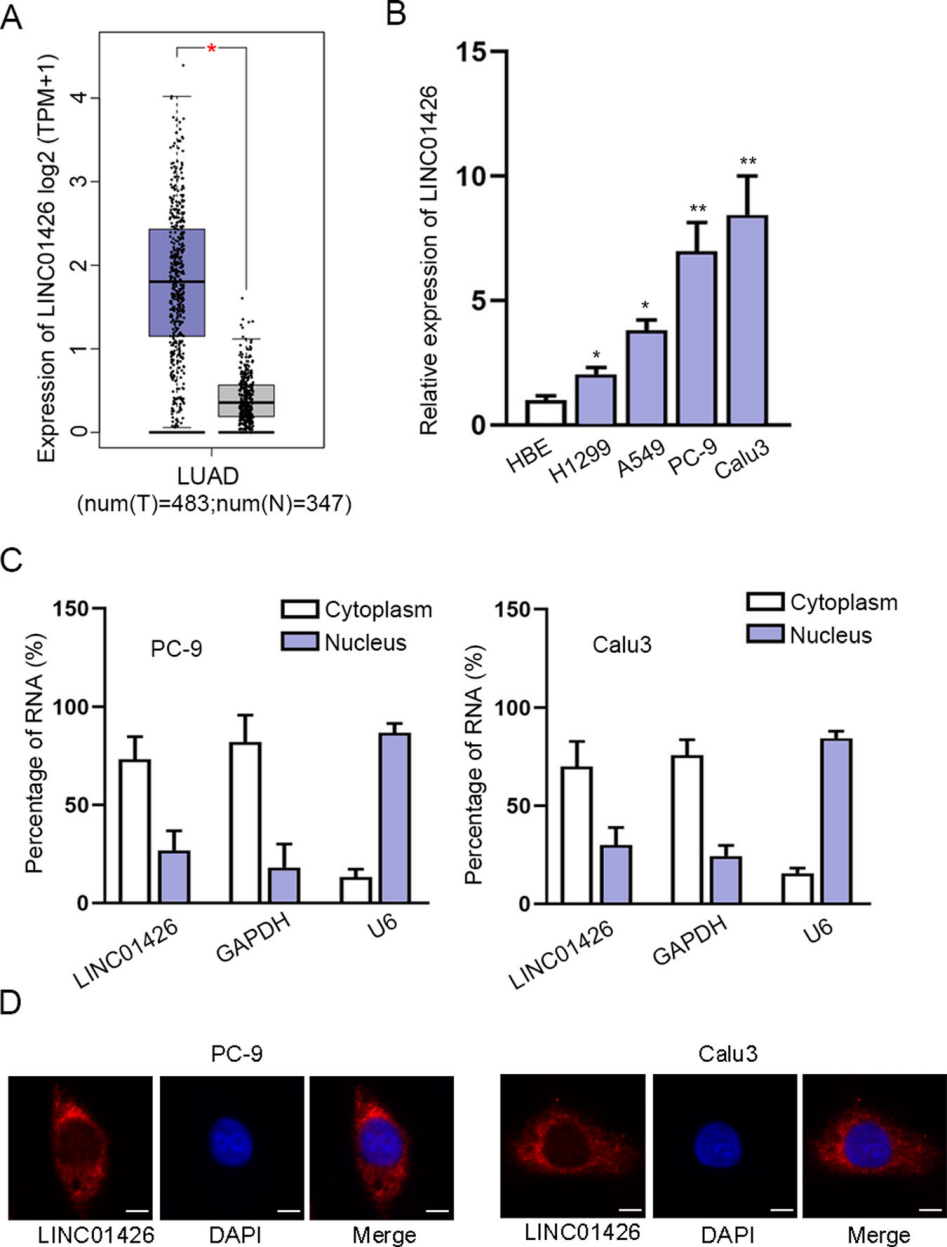
LINC01426 is overexpressed in LUAD. **A** LINC01426 expression in LUAD tissues (left panel; *n* = 483) and normal tissues (right panel; *n* = 347) based on the GEPIA2 database. Unpaired Student’s *t* test. **B** The relative level of LINC01426 in LUAD cells was measured using RT-qPCR. One-way ANOVA. **C**, **D** Subcellular fractionation assay and FISH assay analyzed the cellular location of LINC01426. ^*^P < 0.05, ^**^P < 0.01.

At first, we searched on the GEPIA database (http://gepia2.cancer-pku.cn/#analysis) and found that LINC01426 was expressed at a high level in 483 LUAD tissues compared to 347 normal tissues (Fig. 1A). In addition, we used RT-qPCR to confirm that LINC01426 expression was evidently increased in LUAD cell lines (H1299, A549, PC-9, and Calu3) compared to HBE cell line HBE (Fig. 1B). We selected PC-9 and Calu3 cells for subsequent experiments due to the expression of LINC01426 was expressed highest in these two cells. Furthermore, the Subcellular fractionation assay and FISH assays analyzed that LINC01426 was mainly distributed in the cytoplasm (Fig. 1C, D). Taken together, LINC01426 is highly expressed in LUAD samples and cells.

### Silencing of LINC01426 inhibits proliferation and promotes apoptosis of LUAD cells

**Fig. 2:**
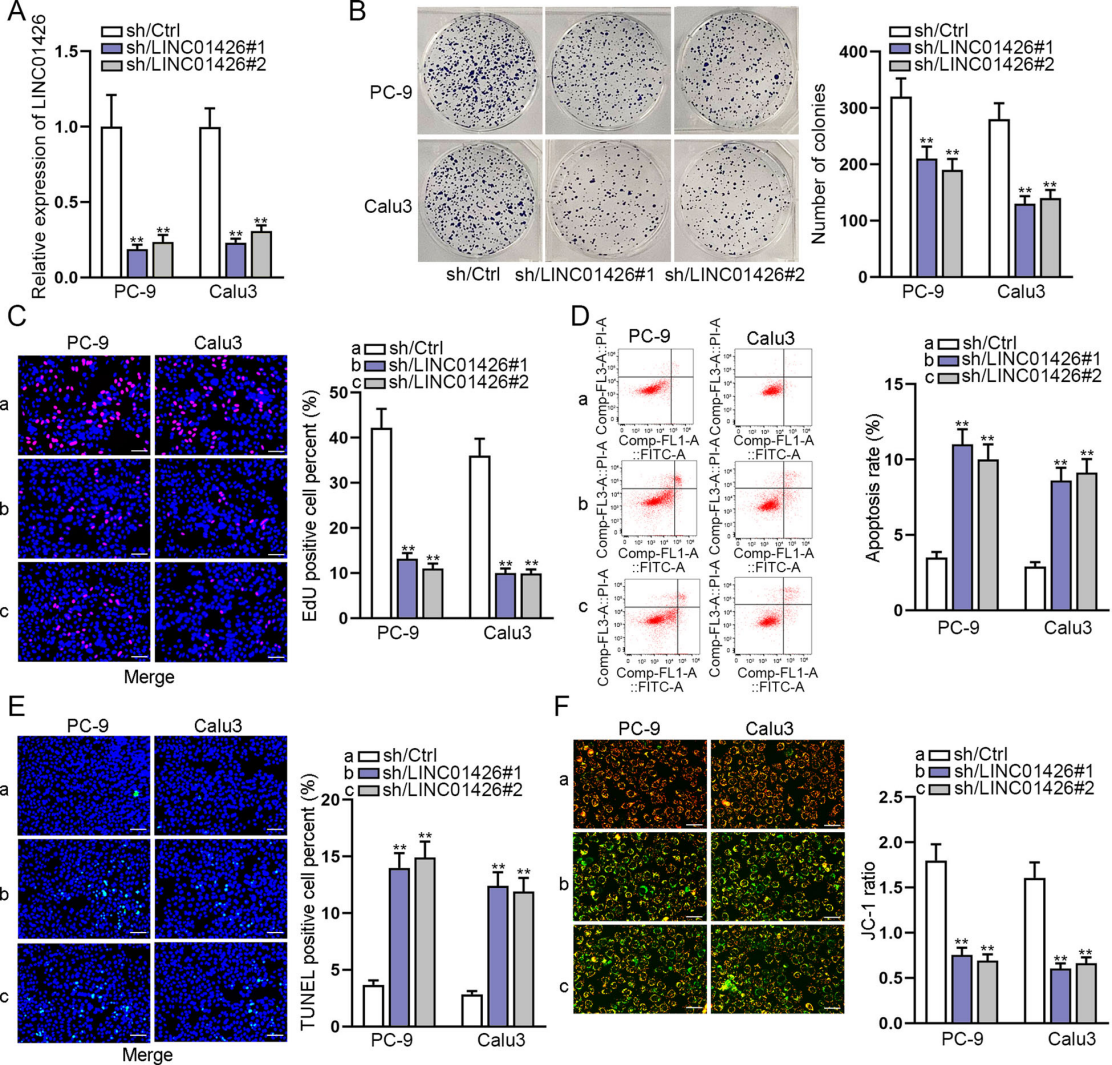
Silencing of LINC01426 inhibits cell proliferation and promotes apoptosis of LUAD cells. **A** Knockdown efficiency of LINC01426 was evaluated using RT-qPCR. One-way ANOVA. **B**, **C** The proliferation of PC-9 and Calu3 cells with LINC01426 silencing was assessed using colony formation assay and EdU assay. One-way ANOVA. **D**–**F** The apoptosis rate of PC-9 and Calu3 cells were evaluated by flow cytometry analysis, TUNEL assay, and JC-1 assay. One-way ANOVA. ^**^P < 0.01.

LINC01426 expression was silenced in PC-9 and Calu3 cells to explore the functions of LINC01426 in LUAD cells (Fig. 2A). It was revealed that the proliferation of PC-9 and Calu3 cells was drastically inhibited by LINC01426 knockdown through colony formation and EdU assays (Fig. 2B, C). Moreover, flow cytometry analysis, TUNEL assay, and JC-1 assay demonstrated that the apoptosis rate of PC-9 and Calu3 cells were increased by LINC01426 knockdown (Fig. 2D–F). All these results showed that silencing of LINC01426 inhibits LUAD cell growth.

### LINC01426 knockdown suppresses migration, EMT, and stemness of LUAD cells

**Fig. 3:**
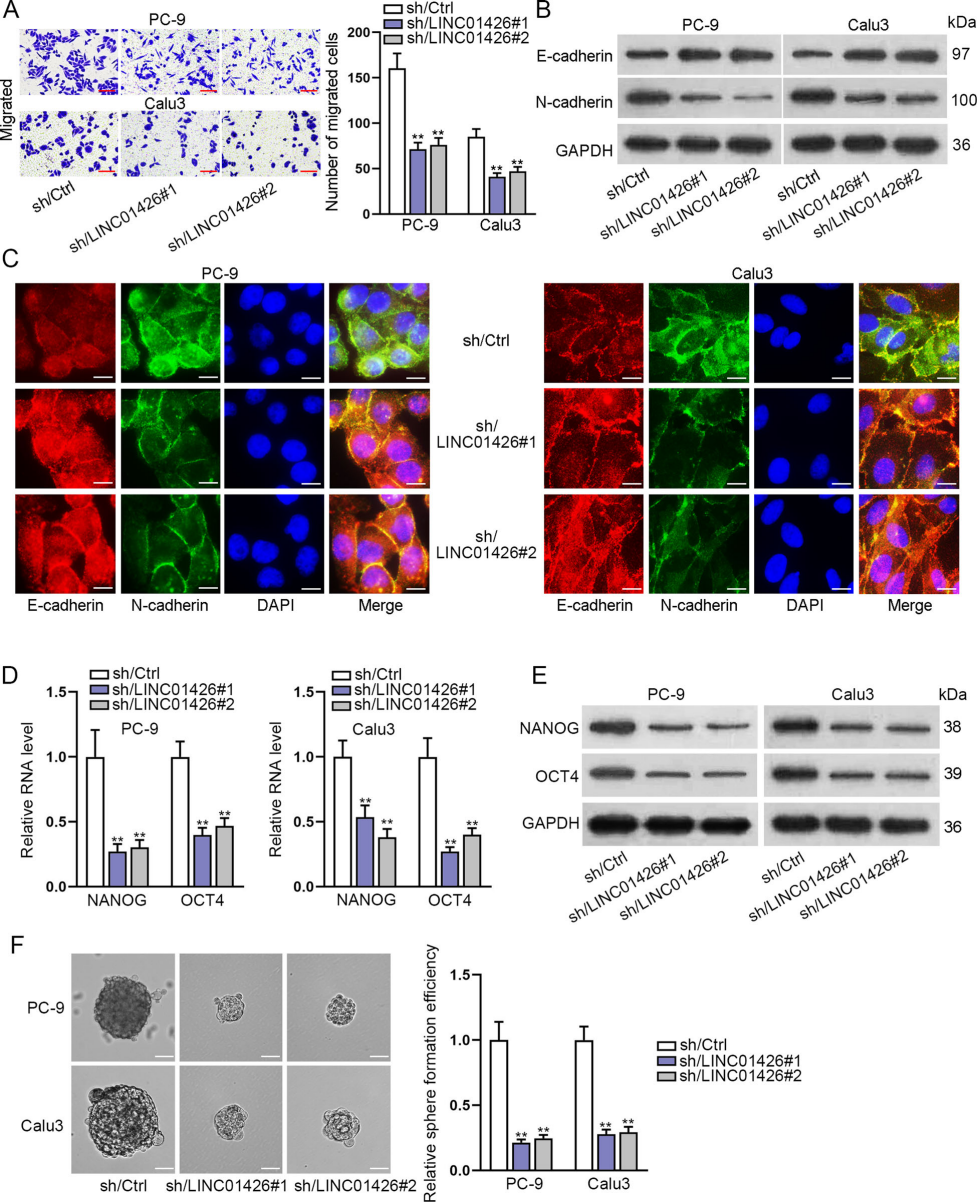
LINC01426 knockdown suppresses migration, EMT, and stemness of LUAD cells. **A** Migration of PC-9 and Calu3 cells were assessed using a transwell assay after transfection with LINC01426-specific shRNAs. One-way ANOVA. **B**, **C** The relative protein levels of E-cadherin and N-cadherin were measured using western blot analysis and immunofluorescence assay. **D**, **E** The RNA level and proteins of NANOG and OCT4 were measured by RT-qPCR and western blot. One-way ANOVA. **F** Sphere formation assay was performed to detect the number of spheres of LINC01426-silenced LUAD cells. One-way ANOVA. ^**^P < 0.01.

We further determine whether LINC01426 could regulate other cell functions in LUAD. Transwell assay certified that LINC01426 knockdown suppressed the migration of PC-9 and Calu3 cells (Fig. 3A). Western blot analysis and IF assays revealed that LINC01426 knockdown increased the expression of E-cadherin while decreased N-cadherin expression in LUAD cells (Fig. 3B, C). Furthermore, the mRNA and protein levels of NANOG and OCT4 (stemness markers) were reduced by LINC01426 knockdown in PC-9 and Calu3 cells (Fig. 3D, E). Sphere formation assay further certified that LINC01426 knockdown suppressed the sphere formation capacity of PC-9 and Calu3 cells (Fig. 3F). All these results indicated that LINC01426 knockdown suppresses the migration, EMT, and stemness of LUAD cells.

### LINC01426 promotes LUAD progression by activating the hedgehog pathway

**Fig. 4:**
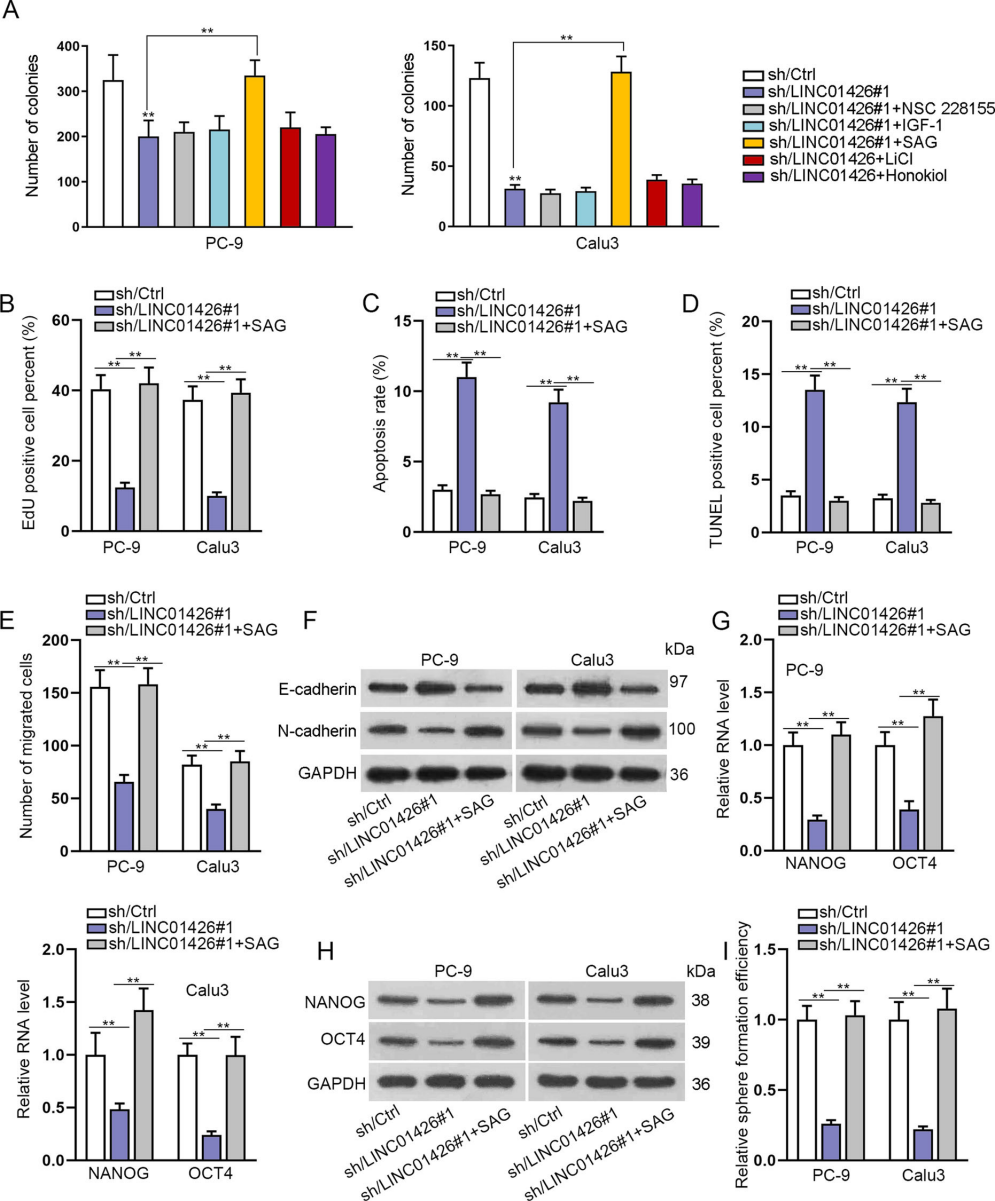
LINC01426 promotes LUAD progression through activating the hedgehog pathway. **A** The number of colonies was analyzed in PC-9 and Calu3 cells treated with sh/Ctrl, sh/LINC01426#1, sh/LINC01426#1+NSC 228155, sh/LINC01426#1+IGF-1, sh/LINC01426#1+SAG, sh/LINC01426#1+LiCl, sh/LINC01426#1+Honokiol, respectively. One-way ANOVA. PC-9 and Calu3 cells were treated with sh/Ctrl, sh/LINC01426#1, or sh/LINC01426#1+SAG and used for subsequent rescue experiments. **B** EdU-positive cells were assessed by EdU assay. One-way ANOVA. **C**, **D** The apoptosis rate was evaluated by flow cytometry analysis and TUNEL assay. One-way ANOVA. **E** The migratory capacity was tested by transwell assay. One-way ANOVA. **F** The relative protein levels of E-cadherin and N-cadherin were detected by western blot. One-way ANOVA. **G**, **H** The RNA and protein levels of NANOG and OCT4 were examined by RT-qPCR and western blot in indicated cells. One-way ANOVA. **I** Sphere formation assay was conducted to test the efficiency of the sphere in indicated cells. One-way ANOVA. ^**^P < 0.01.

LncRNAs have been reported to function in LUAD through various signaling pathways. Here, we added activators of these pathways into PC-9 and Calu3 cells transfected with sh/LINC01426#1 to observe the changes in colony formation ability. The results indicated that the number of colonies reduced by LINC01426 silencing was apparently increased by the treatment SAG activator (Fig. 4A), indicating that LINC01426 functioned in LUAD cells potentially through activating the hedgehog pathway. EdU assay further proved that SAG could completely rescue the EdU positive cells of LINC01426 knockdown in PC-9 and Calu3 cells (Fig. 4B). It was shown that the apoptosis rate enhanced by LINC01426 downregulation was decreased again by SAG (Fig. 4C, D). Furthermore, a transwell assay was performed to disclose that silencing of LINC01426 depleted the migration of PC-9 and Calu3 cells, whereas this effect could be reversed by the treatment of SAG (Fig. 4E). In addition, the increased protein level of E-cadherin and the decreased protein level of N-cadherin caused by LINC01426 silencing were recovered after SAG treatment (Fig. 4F). RT-qPCR and western blot analyzed that the expression of NANOG and OCT4 lessened by LINC01426 knockdown was strengthened by SAG treatment (Fig. 4G, H). Sphere formation assay further asserted that the sphere formation ability of LUAD cells reduced by LINC01426 knockdown was enhanced by SAG treatment (Fig. 4I). In short, LINC01426 exerts functions in LUAD through the hedgehog pathway.

### LINC01426 knockdown enhances the ubiquitination level of SHH protein

**Fig. 5:**
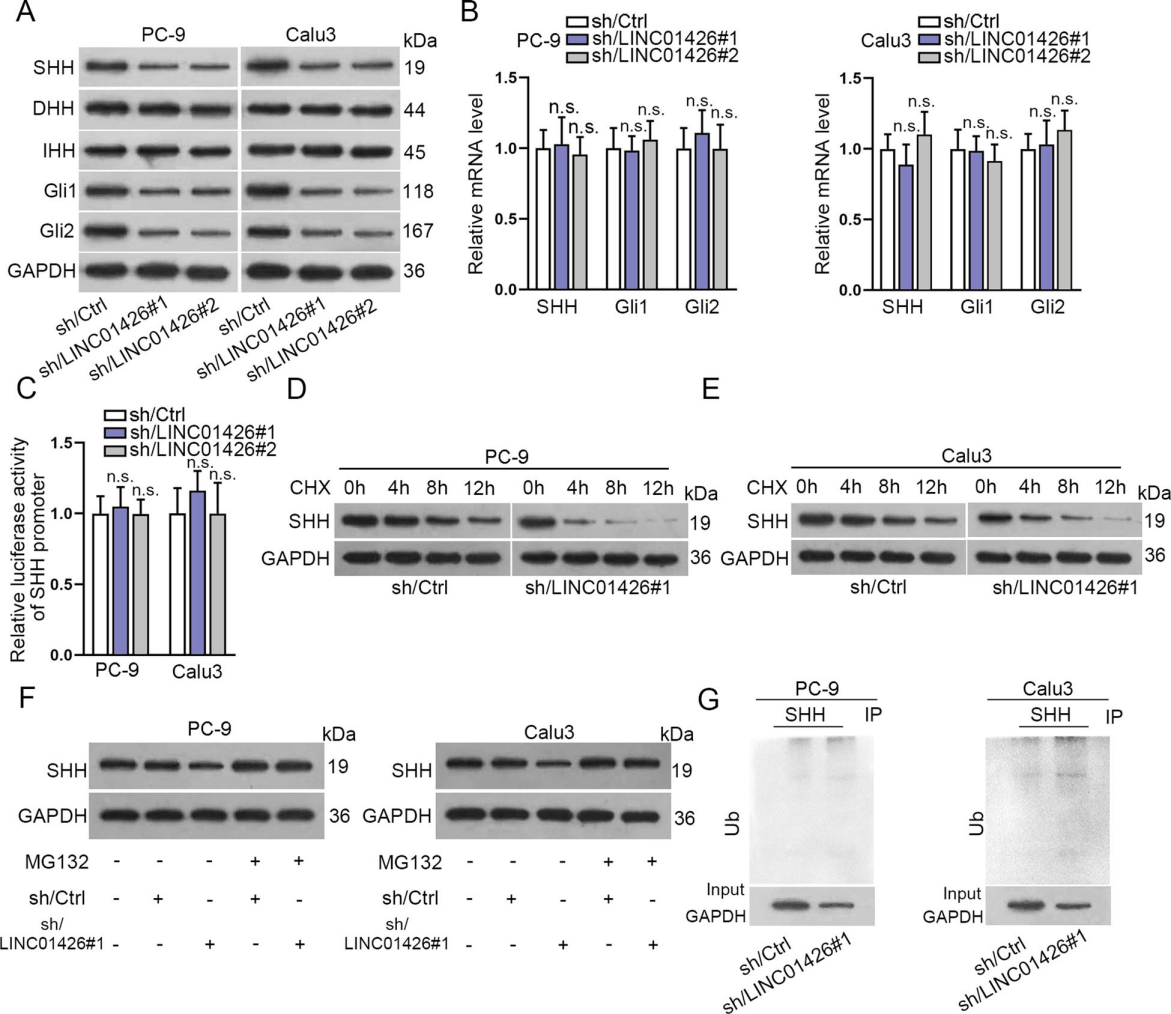
LINC01426 knockdown enhances the ubiquitination level of SHH protein. **A** The relative levels of core proteins in the hedgehog pathway were evaluated in cells with LINC01426 knockdown. **B** The relative mRNA levels of SHH, Gli1, and Gli2 was evaluated by RT-qPCR. One-way ANOVA. One-way ANOVA. **C** The luciferase activity of SHH promoter was determined in cells transfected with shRNAs targeting LINC01426. One-way ANOVA. **D**, **E** The level of SHH protein was measured in PC-9 and Calu3 cells transfected with sh/LINC01426#1 after treatment with CHX for different time points. **F** SHH protein level in PC-9 and Calu3 cells treated with MG132 after transfected with sh/Ctrl or sh/LINC01426#1. **G** The ubiquitination level of SHH protein in PC-9 and Calu3 cells transfected with sh/LINC01426#1 or sh/Ctrl. n.s. no significance.

We further used western blot to analyze the expression of the relative proteins in the hedgehog pathway. As shown in Fig. 5A, the expression of SHH, Gli1, and Gli2 were distinctly reduced by LINC01426 knockdown, while no significant difference between DHH and IHH in PC-9 and Calu3 cells. RT-qPCR examined that the expression of SHH, Gli1, and Gli2 was not changed by LINC01426 knockdown in PC-9 and Calu3 cells (Fig. 5B). Luciferase reporter assay further proved that the luciferase activity of SHH promoter was not changed in PC-9 and Calu3 cells transfected with shRNAs targeting LINC01426 (Fig. 5C), suggesting that LINC01426 could not affect the transcription of SHH. Therefore, we further investigated whether LINC01426 regulated SHH protein. We treated LINC01426-silenced PC-9 and Calu3 cells with CHX to test SHH protein level. Compared with the control group, the half-life of SHH was much shorter in LINC01426-silenced PC-9 and Calu3 cells than that in control cells (Fig. 5D, E and Fig. S1A). SHH protein level was also detected in PC-9 and Calu3 cells transfected with sh/Ctrl or sh/LINC01426#1 treated with or without MG132. It was uncovered that SHH protein level was increased in cells treated with MG132 and this tendency could not be changed by LINC01426 silencing (Fig. 5F). Thus, we hypothesized that LINC01426 might affect SHH protein decay. Ubiquitination experiments further validated that silencing of LINC01426 promoted the ubiquitination of SHH (Fig. 5G). Totally, LINC01426 knockdown regulates the ubiquitination of SHH protein and thus activates the hedgehog pathway.

### LINC01426 binds with USP22 to promote SHH deubiquitination

**Fig. 6:**
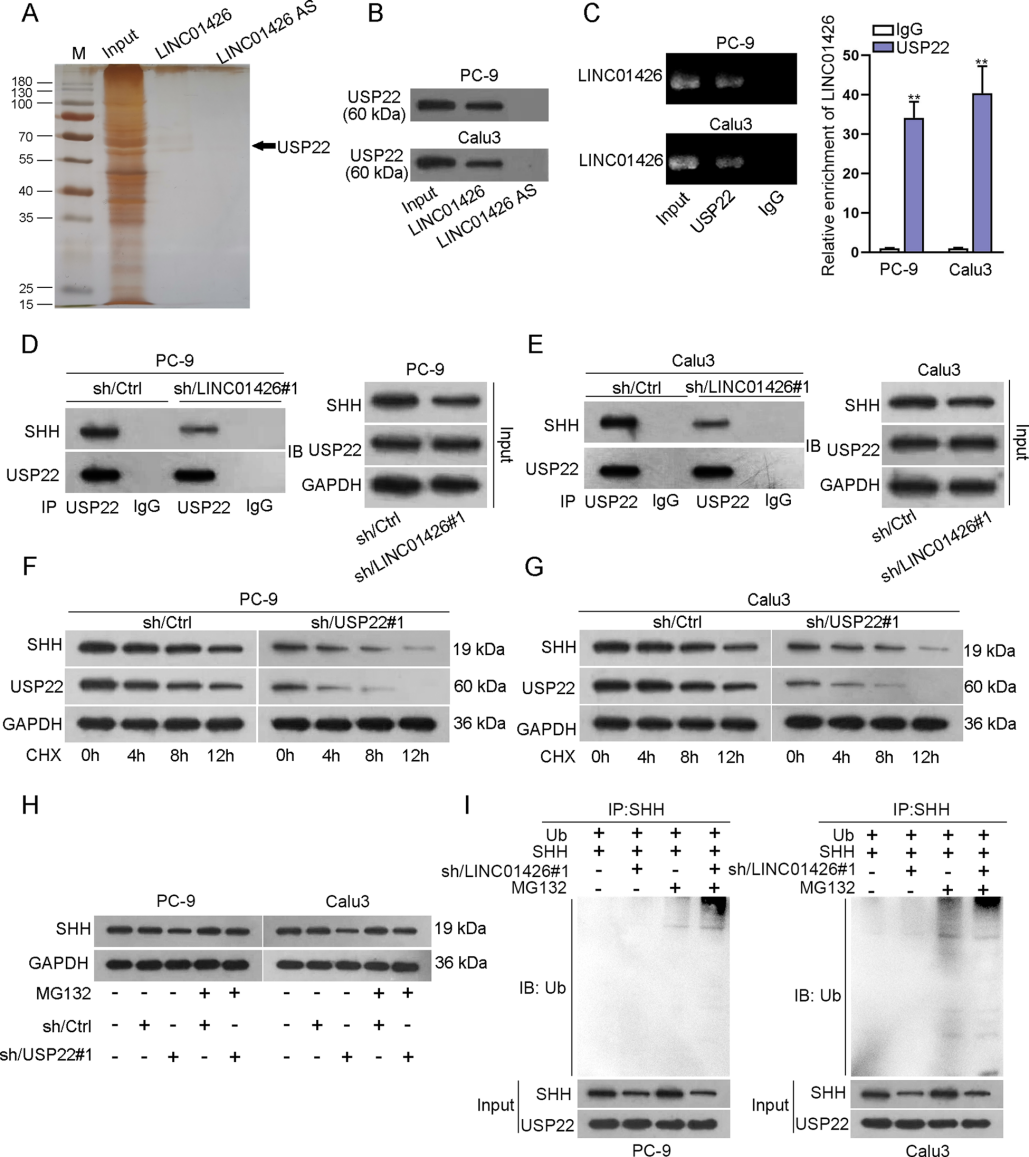
LINC01426 binds with USP22 to promote SHH deubiquitination. **A** Pull-down sliver staining assay and spectrometry analysis assessed the potential proteins combined with LINC01426. **B** Pull-down and western blot analysis revealed the interaction between USP22 and LINC01426. **C** RIP assay examined the enrichment of LINC01426 in the anti-USP22 group and anti-IgG. One-way ANOVA. **D**, **E** The binding of SHH and USP22 was disrupted by LINC01426. **F**, **G**. The level of SHH or USP22 protein was detected in LINC01426-silenced PC-9 and Calu3 cells after treatment with CHX for different time points. **H** SHH protein was detected in USP22-silenced PC-9 and Calu3 cells treated with MG132. **I** Ubiquitination assay was used to detecting SHH ubiquitination in PC-9 and Calu3 cells transfected with sh/LINC01426#1 after treated with MG132. ^**^P < 0.01.

To explore the mechanism by which LINC01426 regulated USP22 protein, pull-down sliver staining was conducted to find potential proteins interacting with LINC01426, and spectrometry analysis confirmed that USP22 was the protein that could closely bind with LINC01426 (Fig. 6A). Pull-down Silver staining followed by western blot analysis further demonstrated the interaction between LINC01426 and USP22 in PC-9 and Calu3 cells (Fig. 6B). RIP assay indicated that LINC01426 was enriched in the anti-USP22 group (Fig. 6C). Through co-IP assay, we determined that the interaction between SHH and USP22 was disrupted by LINC01426 knockdown (Fig. 6D, E and Fig. S1B). According to the correlation analysis of the GEPIA database, there was no significant correlation between LINC01426 and USP22 as well as between USP22 and SHH (Fig. S1C, D). However, LINC01426 and SHH were positively correlated with each other (Fig. S1E). Their interaction was further proven by FISH co-localization analysis (Fig. S1F). Importantly, there no regulatory effect of LINC01426 on USP22 expression (Fig. S1G). Moreover, we silenced in PC-9 and Calu3 cells for further study (Fig. S2A). We applied western blot to demonstrate that the half-life of SHH and USP22 was decreased in PC-9 and Calu3 cells transfected with sh/USP22#1 (Fig. 6F, G and Fig. S2B). Meanwhile, the protein level of SHH enhanced by MG132 was not changed by USP22 silencing (Fig. 6H). Ubiquitination assay revealed that USP22 silencing could promote the ubiquitination level of SHH (Figure S2,C). Ubiquitination assays manifested that downregulation of LINC01426 increased SHH ubiquitination in PC-9 and Calu3 cells supplemented with MG132 (Fig. 6I). Collectively, LINC01426 can recruit USP22 to promote SHH deubiquitination.

### SHH overexpression reverses the inhibitory effect of LINC01426 knockdown on LUAD cell proliferation, migration, EMT, and stemness

**Fig. 7:**
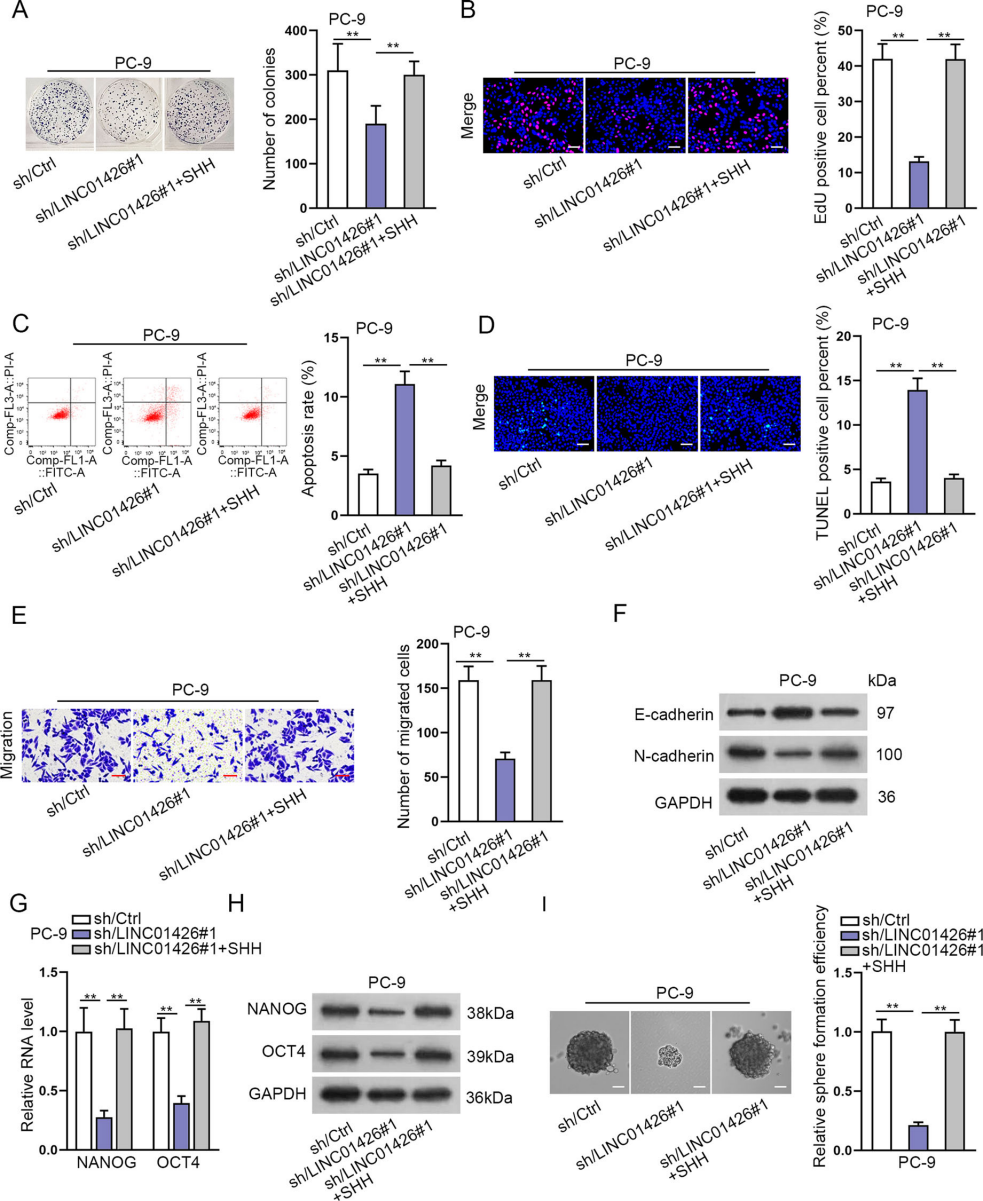
SHH overexpression reverses the inhibitory effect of LINC01426 knockdown on LUAD cell proliferation, migration, EMT, and stemness. **A**, **B**. The proliferation in PC-9 cells transfected with sh/LINC01426#1 or sh/LINC01426#1+SHH was measured using colony formation and EdU assays. One-way ANOVA. **C**, **D** The apoptosis rate was determined in PC-9 cells transfected with sh/Ctrl, sh/LINC01426#1 or sh/LINC01426#1+SHH by flow cytometry analysis and TUNEL assay. One-way ANOVA. **E** The number of migrated cells was determined in PC-9 cells transfected with sh/Ctrl, sh/LINC01426#1, or sh/LINC01426#1+SHH by transwell assay. One-way ANOVA. **F** The protein levels of E-cadherin and N-cadherin were analyzed in PC-9 cells transfected with sh/Ctrl, sh/LINC01426#1, or sh/LINC01426#1+SHH using western blot analysis. One-way ANOVA. **G**, **H** The relative RNA and protein levels of stemness markers were detected by RT-qPCR and western blot in indicated PC-9 cells. One-way ANOVA. **I** Sphere formation assay detected the number of spheres in PC-9 cells. One-way ANOVA. ^**^P < 0.01.

To explore the role of SHH in the LUAD cells, we overexpressed SHH in PC-9 cells (Fig. S3A) and conducted rescue assays. It was displayed in colony formation and EdU assays that SHH overexpression reversed the suppressive effect of LINC01426 knockdown on cell proliferation (Fig. 7A, B). Furthermore, the apoptosis rate enhanced by LINC01426 silencing was decreased again after overexpression of SHH (Fig. 7C, D). Transwell assay revealed the number of migrated cells reduced by LINC01426 knockdown was increased again by SHH promotion (Fig. 7E). The effect of LINC01426 downregulation on EMT-related proteins was attenuated by SHH overexpression (Fig. 7F). Furthermore, the stemness weakened by LINC01426 silencing was strengthened again by the upregulation of SHH of NANOG, and OCT4 was reduced by LINC01426 reduction, and completely overset by overexpressed SHH (Fig. 7G–I).

In vivo experiments were also conducted to validate the role of the LINC01426/SHH axis in LUAD tumor growth. Tumor size, volume, and weight in the sh/LINC01426#1 group were smaller than those in the sh/Ctrl group, whereas, all those indexes were amplified in the sh/LINC01426#1+SHH group (Fig. S3B–D). We also examined the expression level of LINC01426, USP22, and SHH in three groups. It was uncovered that the levels of LIN01426 were decreased in the sh/LINC1426#1 group but were not changed in the sh/LINC01426#1+SHH group. The level of USP22 in the three groups showed no significant difference. However, the level of SHH decreased by LINC01426 silencing was enhanced again after introduction with SHH expression vector (Fig. S3E). IHC assay revealed that the positivity of Ki-67 and PCNA decreased by LINC01426 was enhanced after overexpression of SHH (Fig. S3F).

**Fig. 8:**
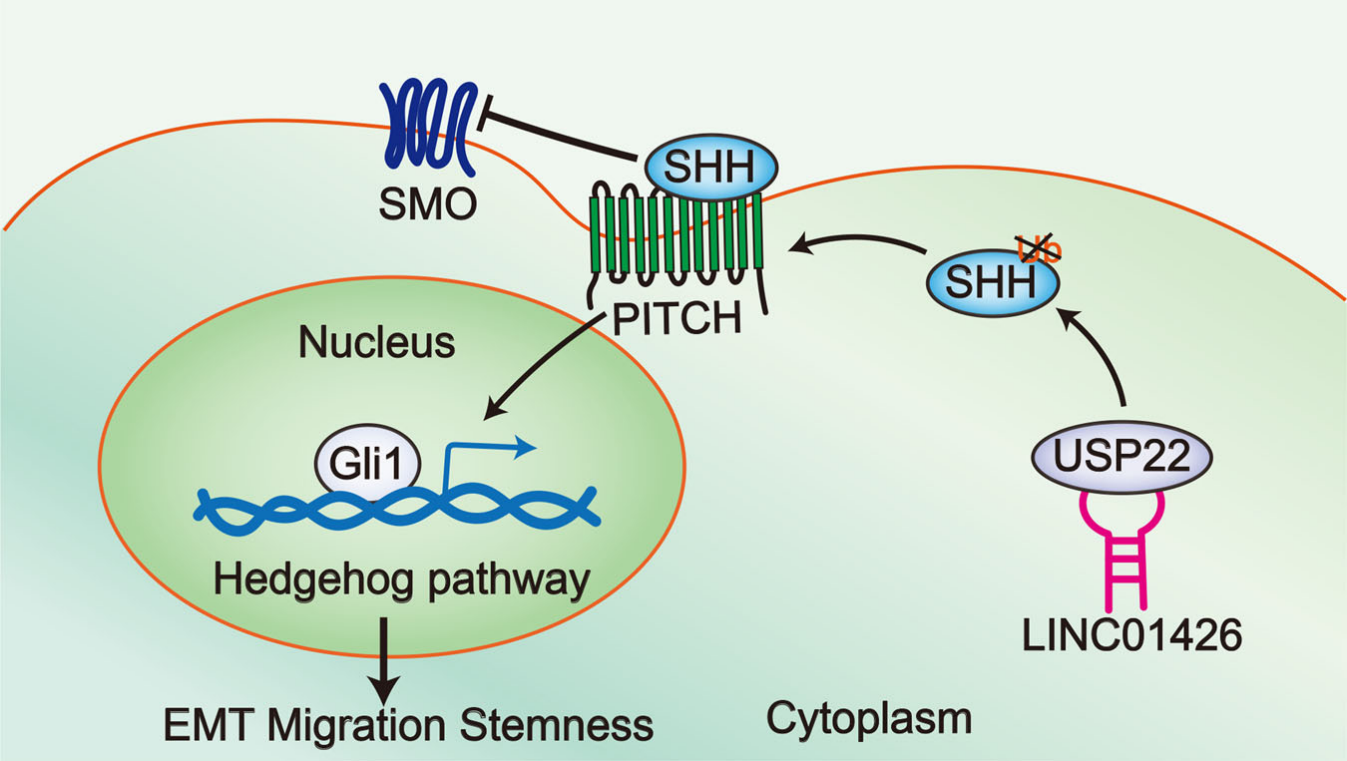
A scientific diagram was generated to illustrate the role of LINC01426 in modulating the LUAD cellular process through the hedgehog pathway.

All these results showed that LINC01426 facilitates LUAD progression via deubiquitination of SHH protein to activate the hedgehog pathway (Fig. 8).

## Discussion

Mounting evidence has revealed that lncRNAs play vital roles in the progression of LUAD. For instance, LINC01614 promotes LUAD cell progression by miR-217/FOXP1 axis^24^. LncRNA CAR10 enhances LUAD metastasis through miR-203/30/SNAI axis^25^. LncRNA SPRY4-IT1 plays important role in tumor cell migration and invasion of LUAD^26^. However, the association between LINC01426 and LUAD was uncovered. In our study, the expression of LINC01426 was found to be dramatically upregulated in LUAD cells. Through loss-of-function assays, we found that LINC01426 knockdown suppressed LUAD cell proliferation and migration. Moreover, EMT and stemness were important processes in LUAD development. We found that silencing of LINC01426 also significantly inhibited the EMT and stemness of LUAD cells. Together, our study suggested that LINC01426 might play an oncogenic role in LUAD, which might be an underlying therapeutic target in LUAD.

To explore the mechanism of LINC01426-regulated LUAD cellular processes, our research further studied the potential underlying signaling pathways. Recently, it has been reported that abnormal activation of multiple signaling pathways is important to LUAD progression. For example, LPCAT1 regulates LUAD metastasis by activating PI3K/AKT/MYC pathway^27^. NRG1 suppresses human lung adenocarcinoma through AKT and ERK1/2 pathway^28^. FLOT1 affects the progression of lung adenocarcinoma by regulating the Erk/Akt signaling pathway^29^. In our study, we observed that LINC01426 functioned in LUAD cells through the hedgehog pathway. Hedgehog pathway plays momentous roles in many malignant tumors, such as hepatocellular carcinoma^30^, medulloblastoma^31^, glioblastoma^32^, and pancreatic cancer^33^. Through further investigation, we found that LINC01426 knockdown inhibited the protein level of SHH but had no significant effect on its mRNA level. Moreover, LINC01406 could not affect SHH transcription. Therefore, we explored whether LINC01426 regulated the stability of SHH protein.

USP22 has been reported to involve in tumorigenesis and progression. For example, MiR-101 impedes the tumorigenesis of papillary thyroid carcinoma by targeting USP22^34^. MiR-30e-5p targets USP22 to suppress the proliferation of nasopharyngeal carcinoma cells^35^. Silencing of USP22 promotes human retinoblastoma cell apoptosis by inhibiting TERT/P53 pathway^36^. Here, we uncovered that LINC01426 could interact with USP22 in LUAD cells. It has been reported that ubiquitination plays vital roles in a variety of biological processes, including cell survival and differentiation as well as innate and adaptive immunity^37^. LncRNAs can interact with ubiquitination-related proteins to modulate the protein level of their downstream targets. For instance, lncRNA uc.134 inhibits CUL4A-mediated ubiquitination of LATS1 to hinder hepatocellular carcinoma progression^38^. LncRNA MEG3 suppresses the progression of gallbladder cancer by promoting the ubiquitination of EZH2 to regulate LATS2^39^. USP22 is known as a deubiquitinase. Here, we determined that LINC01426 interacted with USP22 to stabilize SHH protein.

In summary, our study demonstrated the role of LINC01426 in LUAD progression. LINC01426 knockdown impeded the progression, migration, EMT, and stemness of LUAD cells. In addition, upregulation of LINC01426 promotes LUAD progression by enhancing the level of SHH protein to activate the hedgehog pathway. Our findings may contribute to LINC01426 may serve as a potential therapeutic target in LUAD. Lacking clinical study is a shortcoming of the current study. We will probe the clinical significance of LINC01426 for LUAD patients.

## Supplementary information

Supplementary figure legends

Figure S1

Figure S2

Figure S3
