# Supplementary material for: Upregulation of LINC01426 promotes the progression and stemness in lung adenocarcinoma by enhancing the level of SHH protein to activate the hedgehog pathway
Source: Cell Death Dis. 2021 Feb 10;12(2):173. doi: 10.1038/s41419-021-03435-y (PMC7875967; doi:10.1038/s41419-021-03435-y)
Supplement: Supplementary file 1 — Supplementary figure legends [file 41419_2021_3435_MOESM1_ESM.docx]

**Figure S1.** (A) The quantification data for Figure 5D-E. (B) Flag-tagged co-IP assay revealed the effect of LINC01426 silencing on the interaction between USP22 and SHH. One-way ANOVA. 0, 1, 2, 4 indicated the concentration of flag-USP22. (C) The correlation between LINC01426 and USP22. (D) The correlation between USP22 and SHH. (E) The correlation between LINC01426 and SHH. (F) The interaction between LINC01426 and USP22 was determined by FISH co-localization analysis. (G) The mRNA and protein levels of USP22 in cells with LINC01426 knockdown. One-way ANOVA. ^*^P<0.05, ^**^P<0.01.

**Figure S2.** (A) The expression of USP22 was assessed in PC-9 and Calu3 cells transfected with shRNAs targeting USP22 using RT-qPCR and western blot. One-way ANOVA. (B) The quantification data for Figure 6F-G. One-way ANOVA. (C) Ubiquitination assay detected the ubiquitination level of SHH in cells transfected with sh/USP22#1. ^**^P<0.01.

**Figure S3.** (A) SHH expression was detected in PC-9 cell transfected with pcDNA/SHH using RT-qPCR. Student’s t test. (B) Tumor size in three groups of mice injected with cells stably transfected with sh/Ctrl, sh/LINC01426#1 or sh/LINC01426#1+SHH. (C-D) Tumor volume (two-way ANOVA) and tumor weight (one-way ANOVA.) in three groups. (E) The RNA levels of LINC01426, USP22 and SHH in three groups. One-way ANOVA. (F) IHC staining using anti-Ki-67 and anti-PCNA in tumor tissues collected from three groups of mice. ^**^P<0.01.
