# Supplementary figures and images for: Upregulation of LINC01426 promotes the progression and stemness in lung adenocarcinoma by enhancing the level of SHH protein to activate the hedgehog pathway
Source: Cell Death Dis. 2021 Feb 10;12(2):173. doi: 10.1038/s41419-021-03435-y (PMC7875967; doi:10.1038/s41419-021-03435-y)

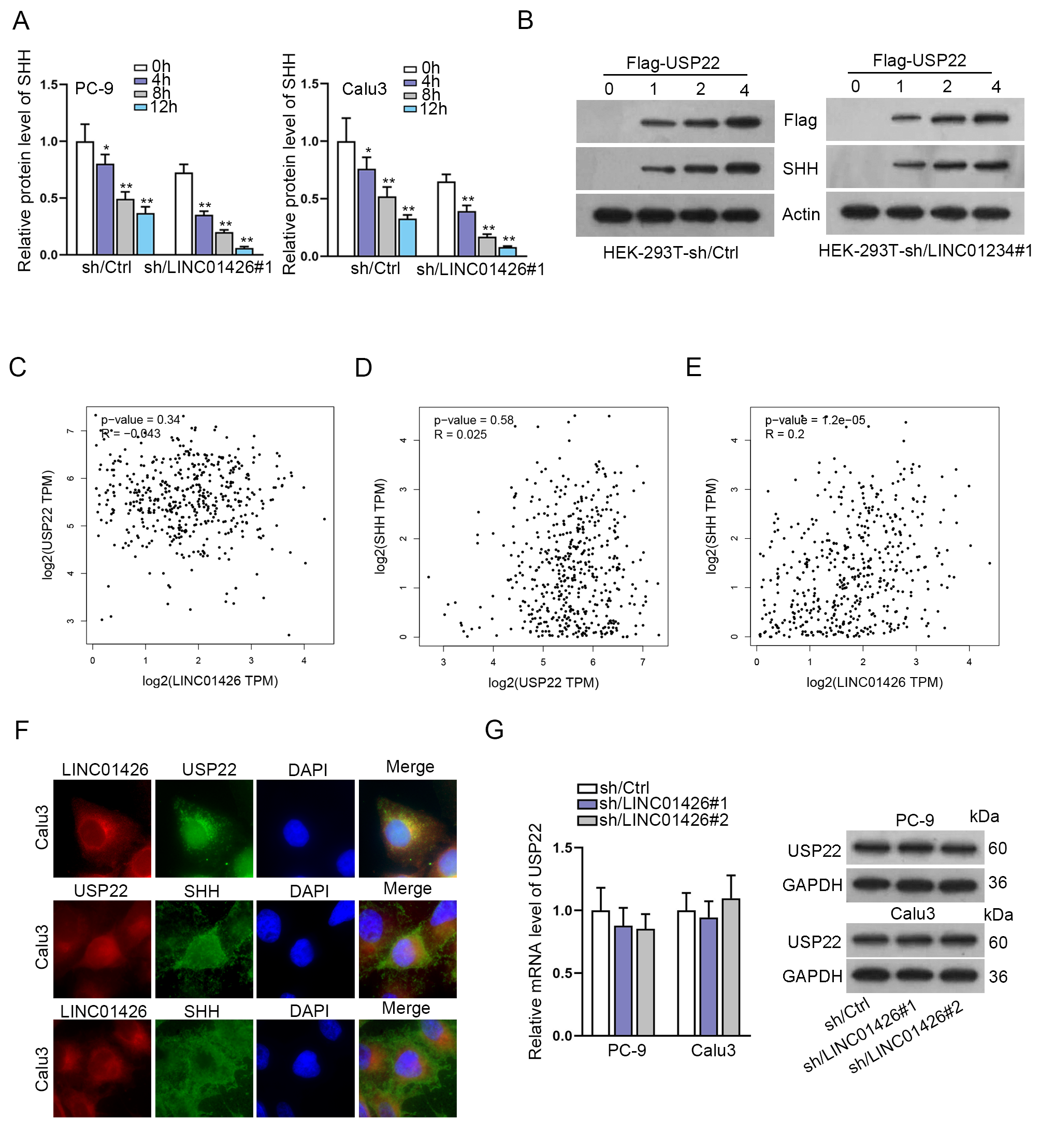

Supplement: Supplementary file 2 — Figure S1 [file 41419_2021_3435_MOESM2_ESM.tif]

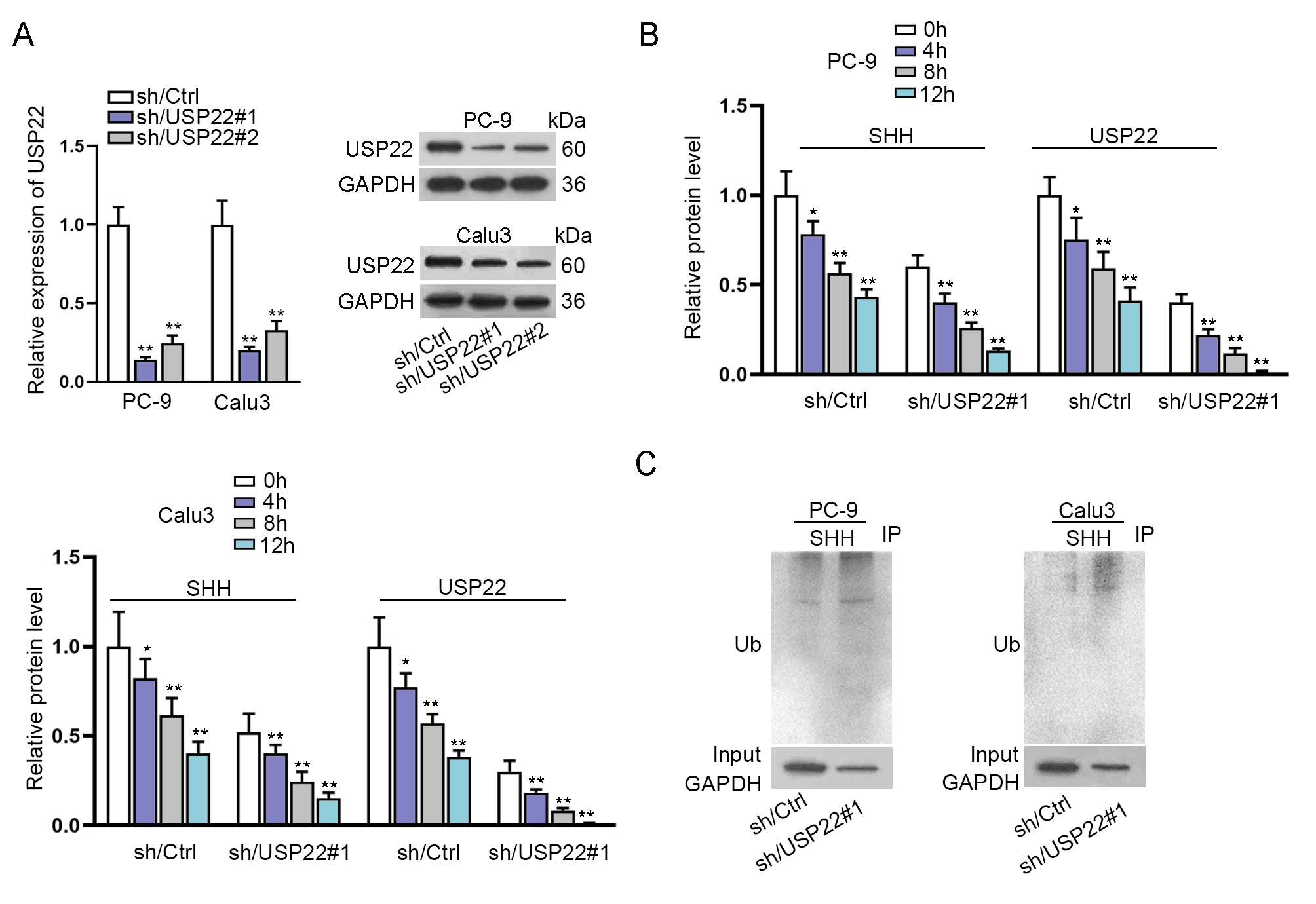

Supplement: Supplementary file 3 — Figure S2 [file 41419_2021_3435_MOESM3_ESM.tif]

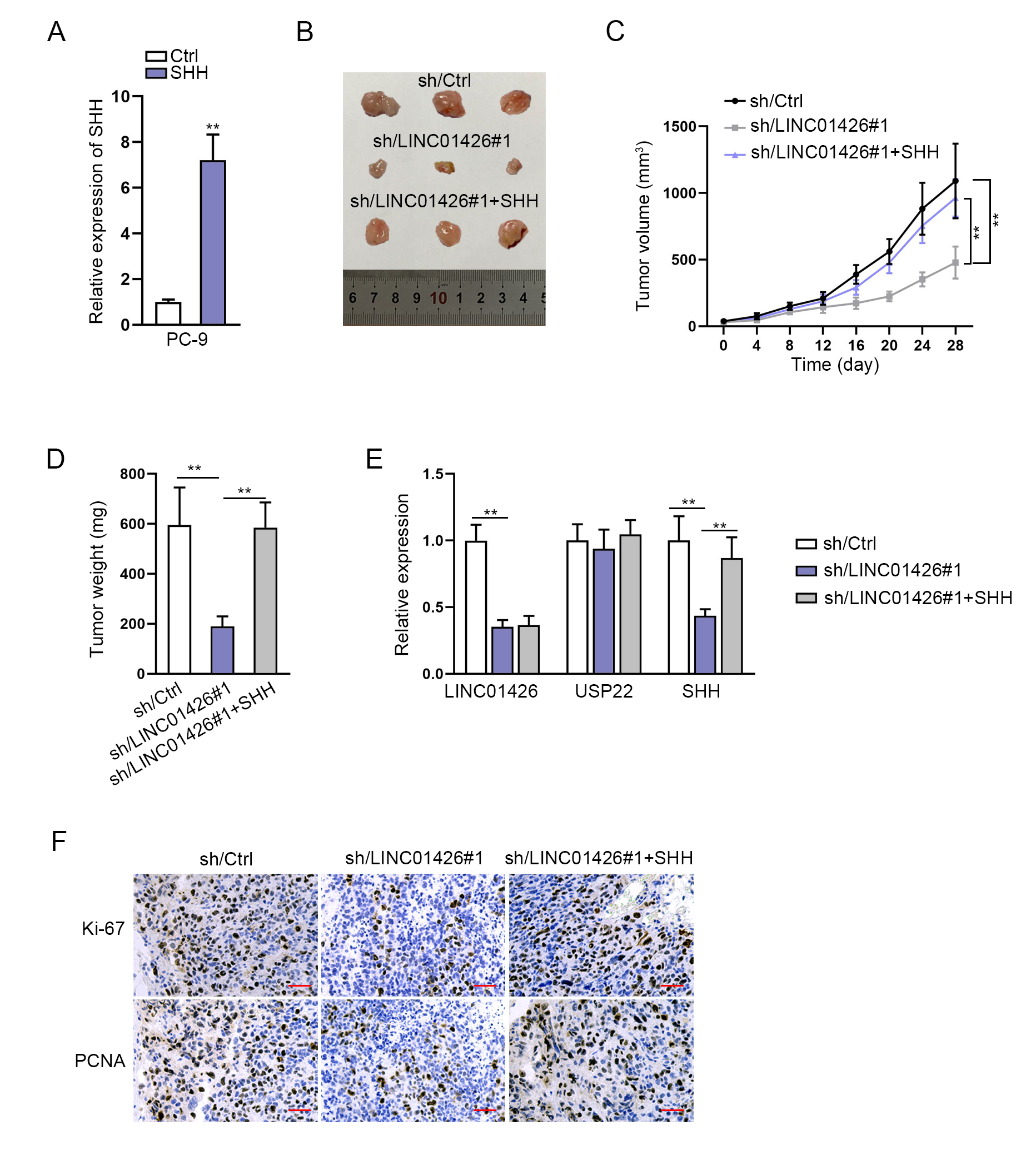

Supplement: Supplementary file 4 — Figure S3 [file 41419_2021_3435_MOESM4_ESM.tif]
